# Supplementary material for: Tead1a Initiates Transcriptional Priming Through the TEAD1a/YAP‐Notch1‐Spi1/Cebpα Axis to Promote Neutrophil Fate
Source: Adv Sci (Weinh). 2025 Aug 28;12(41):e05441. doi: 10.1002/advs.202505441 (PMC12591127; doi:10.1002/advs.202505441)
Supplement: Supplementary file 4 — Supplementary Dataset [file ADVS-12-e05441-s004.zip › 3-6.zip!/3-6/02-6.cluster/cluster16/Enrich/ego_all.pdf]

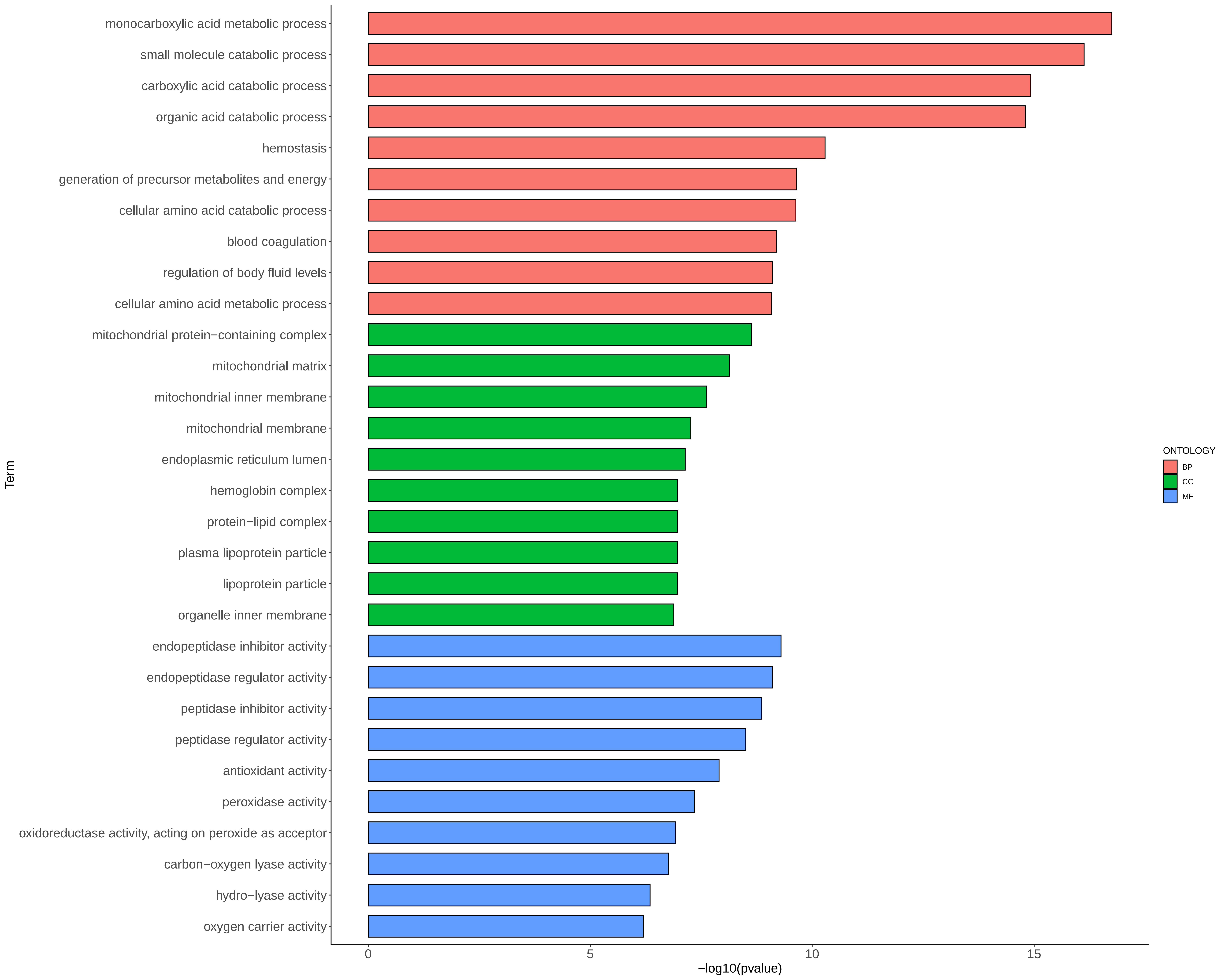

monocarboxylic acid metabolic process
small molecule catabolic process
carboxylic acid catabolic process
organic acid catabolic process
hemostasis
generation of precursor metabolites and energy
cellular amino acid catabolic process
blood coagulation
regulation of body fluid levels
cellular amino acid metabolic process
mitochondrial protein−containing complex
mitochondrial matrix
mitochondrial inner membrane
mitochondrial membrane
endoplasmic reticulum lumen
hemoglobin complex
protein−lipid complex
plasma lipoprotein particle
lipoprotein particle
organelle inner membrane
endopeptidase inhibitor activity
endopeptidase regulator activity
peptidase inhibitor activity
peptidase regulator activity
antioxidant activity
peroxidase activity
oxidoreductase activity, acting on peroxide as acceptor
carbon−oxygen lyase activity
hydro−lyase activity
oxygen carrier activity
Term
0
5
10
15
−log10(pvalue)
ONTOLOGY
BP
CC
MF
